# Functional Organ Preservation Surgery in Head and Neck Cancer: Transoral Robotic Surgery and Beyond

**DOI:** 10.3389/fonc.2019.00293

**Published:** 2019-04-17

**Authors:** Wojciech Golusiński

**Affiliations:** Department of Head and Neck Surgery, Poznan University of Medical Sciences, Poznan, Poland

**Keywords:** TORS, head and neck, organ preservation, robotic surgery, oropharynx

## Abstract

In recent years, interest in functional organ preservation surgery (FOPS) in the treatment of head and neck cancer has increased dramatically as clinicians seek to minimize the adverse effects of treatment while maximizing survival and quality of life. In this context, the use of transoral robotic surgery (TORS) is becoming increasingly common. TORS is a relatively new and rapidly-evolving technique, with a growing range of treatment indications. A wide range of novel, flexible surgical robots are now in development and their commercialization is expected to significantly expand the current indications for TORS. In the present review, we discuss the current and future role of this organ-preserving modality as the central element in the multimodal treatment of head and neck cancer.

## Introduction

Currently, the management of head and neck squamous cell carcinoma (HNSCC) involves a multimodal treatment approach consisting of various combinations of surgery, chemotherapy, and radiotherapy (1, 2). Although the primary aim of treatment is survival, there has been a growing emphasis in recent years on functional outcomes and quality of life (QoL). For this reason, treatment-related effects on physiological function and QoL are crucial considerations in selecting the optimal approach.

While open surgery and concurrent chemoradiotherapy (CRT) both provide good oncological outcomes (3–6), these modalities frequently induce severe functional morbidity and toxicity. Minimally-invasive surgical treatments, including transoral laser microsurgery (TLM) and transoral robotic surgery (TORS), offer the potential for organ preservation with less functional morbidity than open surgery and les toxicity than CRT. This is especially relevant given the increasing incidence of human papilloma virus (HPV)-positive disease, as these patients tend to be younger and have a better long-term prognosis than those with HPV-negative HNSCC (7). This changing patient profile has strengthened interest in functional organ preservation surgery (FOPS) to improve functional outcomes and QoL in these patients.

In this context, the aim of the present review is to explore the evolving and increasingly important role of TORS in the multimodal treatment of HNSCC. We also discuss the future of TORS, in which the development of smaller, more flexible surgical robots is expected to expand the indications for this minimally-invasive approach in the near future.

## Methods

We searched MEDLINE for original and review articles published up to December 2018. The following search terms were used “transoral,” “robotic surgical procedures,” “robot-assisted,” “TORS,” “radiotherapy, intensity-modulated,” “oropharyngeal neoplasms,” and “squamous cell carcinoma.” Only articles on human subjects and published in English were evaluated. The same terms were used to search clinicaltrials.gov to identify relevant clinical trials.

### Functional Organ-Preservation Approaches

A wide range of surgical and non-surgical approaches are available to achieve functional organ preservation in HNSCC (8). Moreover, as we discuss below, numerous randomized clinical trials are currently underway to evaluate different multimodal approaches to functional organ preservation in patients with HNSCC. Non-surgical approaches include mainly chemotherapy and radiotherapy (9). Many different surgical approaches are available, including open surgery, minimally-invasive techniques such as key-hole surgery, and of course transoral techniques (10). However, the specific techniques will depend on the location of the tumor and other patient- and tumor-related factors. The main transoral techniques are TLM and TORS, which we discuss in greater detail below.

### TORS: A Brief History

Robotic surgery was first used in 1985 (11). The first application of robotic surgery in head and neck cancer occurred 20 years later in 2005 when McLeod and Melder used the da Vinci robot to resect a vallecular cyst (12). In 2007, Weinstein et al. reported results of a clinical trial to evaluate TORS for radical tonsillectomy (13). TORS was first approved by the United States Food and Drug Administration (FDA) in 2009 for the treatment of early-stage oropharyngeal cancer. Since then, the indications for TORS in HNSCC have rapidly expanded to include not only the oropharynx, but also the hypopharynx, parapharyngeal space, and supraglottic larynx.

The aim of TORS and other minimally-invasive surgical approaches is to maximize exposure while minimizing surgical morbidity such as tracheostomy, pharyngotomy, and flap reconstruction. However, not all patients are suitable candidates for TORS, most notably due to a lack of adequate endoscopic access. Rich et al. identified eight factors (the eight “Ts”) necessary to ensure proper endoscopic access in patients undergoing TLM: teeth, trismus, transverse dimensions (mandibular), tori, tongue, tilt, treatment (prior radiation), and tumor (14). These same criteria are widely applicable to patient selection for TORS. Other exclusion criteria for TORS include morbid obesity, micrognathia, microstomia, craniofacial abnormalities, and other factors that can prevent robotic access (9, 15–17).

Numerous studies have evaluated the safety and effectiveness of TORS, although long-term data are scant and no randomized trials have yet been conducted to compare TORS to definitive CRT or open surgery (18). As a result, the current indications for TORS are primarily based on retrospective data (1, 2). Although TORS has been used to treat various tumor localizations in head and neck cancer (19–21), the strongest data with the longest history is for the treatment of oropharyngeal cancer, as we discuss below.

### TORS for Oropharyngeal Cancer

The first reports of robotic surgery to treat oropharyngeal cancer were described by Hockstein et al. in a cadaver model (22) and by O'Malley et al. (23). In 2009, the first large prospective case series of TORS to treat oropharyngeal squamous cell carcinoma (OPSCC) was reported by Moore et al. (24) in a study involving 45 patients with stage T1-T4a disease. That study found no major treatment-related complications, leading the authors to conclude that TORS was safe with acceptable oncological outcomes. Subsequently, in the year 2012, Weinstein et al. (25) reviewed the early results from clinical trials conducted at three different hospitals, primarily involving patients with OPSCC. Echoing the conclusions of Moore and colleagues, those authors also concluded that TORS appears to be safe and feasible in the multidisciplinary management of head and neck cancer.

Despite the lack of randomized trials, recent data suggest that the oncological outcomes of TORS for OPSCC is comparable to open surgery and CRT (26). Recently, Moore et al. reported results from a large series (*n* = 314) of patients with OPSCC. At 5-years, the locoregional recurrence-free survival rate was 92%, with an overall survival rate of 86% (27). The largest study of TORS published to date was the international multi-institutional review of 410 patients (mostly early–stage OPSCC) carried out by de Almeida et al. (20). In that study, 3-year survival and recurrence rates were 92.5 and 88.8%, respectively, results that are comparable to those achieved with definitive radiotherapy (3).

Despite the growing popularity of TORS in the treatment of OPSCC, this approach has several potential drawbacks. Importantly, the use of TORS does not obviate the need for postoperative radiotherapy in many cases. Moreover, as Mendenhall et al. (28) have observed, many patients treated with TORS undergo neck dissection, which is not typically necessary if definitive CRT had been used. In addition, like most treatments, TORS can have important treatment-related adverse effects. The most common and serious complication of TORS is postoperative hemorrhage, with an incidence rate ranging from 3 to 8% (16, 29, 30). This potentially fatal complication frequently requires a second surgical procedure to control the bleeding. Nonetheless, emerging data suggest that postoperative bleeding can be reduced—although not avoided—by ligating the ipsilateral external carotid artery (31).

While the initial indications for TORS were in early-stage OPSCC, more recently these indications have expanding to include late-stage disease and even laryngeal and hypopharyngeal cancer (32, 33), in part due to the growing emphasis on organ preservation and QoL.

### TORS for Laryngeal and Hypopharyngeal Cancer

A growing number of studies have reported success with TORS for laryngeal cancer (18, 32), with the most common application of TORS in this context being supraglottic laryngectomy (SGL). Although this region is relatively accessible, access, and maneuverability are limited by anatomic restrictions. In a recent review (18), Gorphe et al. emphasized the importance of completely resecting the tumor while leaving intact the physiological function of the larynx and base of the tongue. For this reason, the indication for TORS-assisted SGL (TORS-SGL) is primarily limited to well-selected patients with early stage (T1-T2) tumors. The first application of TORS for supraglottic cancer (SGL) was described by Weinstein et al. in 2007 (34). Other authors, including Ozer et al. (35) and Mendelsohn et al. (36), have also reported good results with TORS for this indication. In 2016, Razafindranaly et al. (37) reported results of a multicentric study to assess the efficiency, safety, and functional outcomes of TORS-SGL in 84 patients. Overall, outcomes were good, but 9.5% of patients required percutaneous gastrostomy feeding, 24% required a temporary tracheostomy and 1% definitive tracheostomy. In addition, aspiration pneumonia was observed in 23% of the patients (with one death), and postoperative bleeding in 18% of patients. The results of that study led the authors to conclude that TORS is safe and achieves good functional outcomes with fast recovery, but that it also presents a risk of serious adverse events. Based on their findings, the authors emphasized the importance of applying clear selection criteria to reduce the risk of postoperative complications.

TLM is currently considered a standard-of-care for early glottic cancer, but TORS has also been used to treat early-stage glottic carcinomas, although data are limited. The first report of TORS for this indication was made by O'Malley et al. in a canine model (38). Most of the studies conducted to date have involved very small cases series (18), but the early results are promising.

Several reports suggest that TORS can be used for hypopharyngeal cancer, despite the difficult access to this anatomical region. Park et al. (39) compared oncologic and functional outcomes in patients who underwent TORS-assisted hypopharyngectomy (*n* = 30) vs. open surgery (*n* = 26), finding no significant differences in overall and disease-specific survival at 3 years. Relevantly, functional outcomes were better in the TORS group, leading the authors to conclude that TORS hypopharyngectomy is a promising procedure that warrants more study.

### Robot-Assisted Neck Dissection

Some reports have described the use of robotic techniques to perform neck dissection (19, 40), which is especially relevant given the focus on functional outcomes, as these techniques may help to preserve key structures without negatively affecting oncologic outcomes. The most common approach to robotic neck dissection is the retroauricular approach (41), although a transaxillary approach has also been described (42). Most studies of robot-assisted neck dissection have focused on papillary thyroid cancer, reporting similar outcomes to those achieved with open surgery (43). At present, the currently available data for this approach to neck dissection are promising but limited. More studies are needed to determine the safety and oncologic outcomes of this approach and patient suitability.

### Surgical Salvage

In the salvage setting, early evidence suggests that TORS may be superior to open surgery in terms of perioperative and functional outcomes (44, 45), although long-term outcomes from larger studies are needed to better establish the role of TORS in this setting and to more clearly define selection criteria. It is worth underscoring that the decision between different treatment options (e.g., open vs. transoral surgery) is not always black and white, as some patients with recurrent disease may benefit from a hybrid approach (46). Although open surgery has long been the treatment of choice for salvage therapy due to the need for radical resection, TORS may be a viable alternative. Nonetheless, given the relative death of data for this indication, more studies will be needed to clarify the role of TORS in this setting.

### Advantages and Disadvantages of TORS

Given the lack of randomized trials and relatively scant data on long-term oncological outcomes, any discussion of TORS must focus on the available data to provide a clear picture of the advantages and disadvantages of this treatment approach. Transoral approaches have several important theoretical and practical advantages over open surgical techniques, including significantly less cosmetic and functional morbidity, a lower risk of infection, and more rapid recovery. Other benefits of TORS include 3D panoramic vision, 360° range of motion, good optics, hand tremor filtration, and the possibility to perform en bloc resection (47). A key advantage of surgery (both open and transoral) over CRT is the capacity to pathologically evaluate the surgical specimen, thus potentially permitting treatment de-intensification.

Notwithstanding the potential advantages of TORS described above, several important complications have also been reported (48), most notably including postoperative bleeding, a potentially life-threatening complication (21). Reported rates of major postoperative hemorrhage are as high as 9.8% (16, 30). A wide range of other complications have also been reported. In 2018, Hay et al. (49) reviewed data from 122 TORS surgeries performed at the Memorial Sloan Kettering Cancer Center between June 2010 and August 2015, finding a total of 107 complications (66 TORS-related). A major TORS-related complication was observed in 19 patients, including severe complications (grade 4) requiring intensive care treatment such as aspiration with respiratory compromise and hemorrhage. Other severe (grade 3) complications associated with TORS included dysphagia, bleeding, and temporary tracheostomy. Those authors found 24 episodes of postoperative hemorrhage, with 7 cases (5.7%) requiring an invasive intervention. A multicentric French study (50) reported several significant intraoperative complications, including hemorrhage, pharyngeal fistula, and external surgical conversions. Postoperative complications included bleeding, aspiration pneumonia, tracheostomy, pharyngocutaneous fistulae, cervical spondylitis, and even death. These data reflect, in part, the risks of any type of surgery. However, they also reflect the learning curve of TORS. In this regard, Hay et al. found that the complication rate for major TORS-related complications decreased over time, from one-third of patients in 2010 to only 10% in 2015.

### Cost-Effectiveness of TORS

The high cost of robotic systems—which includes not only the robot and related instruments, but also the annual services costs—together with the steep learning curve, and the consequent need for extensive training, make this technique cost-prohibitive for many centers (51). Dombree and colleagues conducted a cost comparison of an open surgical approach, TLM, and TORS in 2014 (52), finding that that TORS was more expensive than standard approaches, mainly due to the purchase and maintenance costs and the use of proprietary instruments.

The high costs of currently-available surgical robots has led some authors argue that the best approach is to centralize the use of TORS in high-volume treatment centers (53) based on studies showing that such centers obtain better outcomes with lower costs than low-volume centers (54–58). A recent study (59) concluded that high surgical volumes are needed to maintain expertise and quality assurance. That same study concluded that TORS is currently underutilized due to the associated expenses (59), particularly in countries with limited resources such as Estonia, Latvia, and Lithuania where TORS is currently unavailable. Although cost is currently an important barrier to the wider uptake of robotic surgery, the impending commercialization of new, smaller, more flexible surgical robots is likely to decrease costs, and expand current indications for TORS (11).

### Transoral Surgery and Treatment de-Intensification

Despite the many advantages of surgery in HNSCC, surgery alone is rarely curative for many cancer types and thus it is important to combine surgery with other therapies—primarily radiotherapy and chemotherapy—to achieve optimal outcomes. In this regard, there is an ongoing change in cancer therapy toward more personalized, multimodality treatment approaches. At present, there is intense research activity underway in an effort to de-intensify treatment in order to reduce long-term toxicity and improve QOL, with numerous large-scale treatment de-intensification trials being carried out (8). The NCT01330056 trial is being carried out to compare FOPS to radiotherapy or (CRT) as the first-line treatment for patients with HNSCC of the oropharynx, larynx, and hypopharynx. Numerous clinical trials are underway to determine if it is possible to de-escalate adjuvant treatment based on pathologic findings from surgery. The ADEPT (Adjuvant De-escalation, Extracapsular Spread, p16 Positive, Transoral) trial (NCT01687413), a phase III trial of HPV-positive, high-risk OPSCC patients treated with transoral surgery with negative margins; the main aim is to determine if postoperative chemotherapy can be omitted. The ECOG 3311, a phase II trial of patients with advanced HPV-positive OPSCC treated with transoral surgery and neck dissection, was completed in July 2017, although the outcomes have not yet been published. The ORATOR trial (The Oropharynx: radiotherapy vs. TORS) is a single-institution trial comparing QOL and survival outcomes in OPSCC treated with transoral surgery or primary radiotherapy. ORATOR2 (NCT03210103) is being conducted to compare the same treatments, but limited to patients with HPV+ OPSCC. The PATHOS trial (Post-operative Adjuvant Treatment for HPV-positive Tumors; NCT02215265) is studying patients with HPV-positive cancer (T1-3, N0-2b) treated by transoral surgery and neck dissection to identify which patients are candidates for treatment de-intensification (60). The EORTC “best of” trial (NCT02984410) is investigating transoral head and neck surgery compared with radiotherapy. The primary and secondary endpoints of this trial are, respectively, swallowing function and overall survival. Finally, another trial (NCT03691441) is designed to determine the effectiveness of transoral head and neck surgery for locally-advanced, transorally-resectable OPSCC followed by risk-adapted adjuvant therapy vs. primary radiochemotherapy.

### Current Controversies

The numerous trials underway underscore the many unresolved questions and controversies regarding the optimal management of patients with HNSCC (1, 3). There are a wide range of treatment options available and the optimal approach is frequently in doubt. The development of advanced radiotherapy techniques such as intensity-modulated radiotherapy (IMRT) and volumetric modulated arc therapy (VMAT) has greatly improved functional outcomes and decreased both early and late toxicity (1, 2). Although the available evidence suggests that TORS provided oncologic outcomes that are compared to primary radiotherapy but with better functional outcomes, it is important to keep in mind the shortcomings of the available evidence. First, as mentioned previously, no randomized trials have been performed to directly compare TORS to definitive CRT (18). In addition, as Yeh et al. observed in their systematic review (1) comparing TORS to radiotherapy for the management of OPSCC, patients with advanced stage disease account for a higher proportion of the sample in studies of IMRT vs. studies that have assessed TORS. Moreover, TORS patients are carefully selected in those studies, which could further bias the results. Yeh et al. also note that most of the current data on TORS comes from high-volume centers and it is not clear to what extent an “expertise” bias may have influenced the oncologic and/or functional outcomes. Finally, it is worth emphasizing that a substantial proportion of patients who undergo TORS also receive adjuvant radiotherapy and/or adjuvant CRT, making it more difficult to disentangle the relative influence of the various treatments on outcomes.

In this regard, the clinical trials described above—together with emerging data from other prospective trials—will help to better determine the true benefit of TORS and to identify patients who are suitable for treatment de-escalation (26, 61, 62). If we can better define these patients, the decrease in adjuvant treatments should improve functional outcomes while also reducing the overall costs of treatment.

### Future Directions: Novel, Flexible Surgical Robots to Overcome Limited Access

Despite the predominance of the da Vinci robotic systems, several novel, flexible surgical robots are currently in development and the commercial introduction of these robots is expected to decrease costs and expand current indications for TORS, thus enabling wider adoption of this technology (11). These tools include new miniaturized, flexible robots, such as the i-Snake® (Imperial College London, London, UK) (63), and Flex® systems (Medrobotics®, Raynham, MA, USA), which are better-suited than the da Vinci robot for head and neck surgery (11). Other companies are also investing in the design and manufacture of new surgical robots, and this new competition is likely to lead to lower costs, making robotic surgery less expensive while, at the same time, increasing the number of indications in locations with difficult access with current technology. Numerous other robotic surgical systems are in development or have recently been introduced into the market (64), including the Versius® surgical robotic system (CMR Surgical, Ltd.; Cambridge, UK) the Senhance TM multi-port and SurgiBot TM single-port robotic platforms (TransEnterix, Inc.; Morrisville, NC, USA) and the SPORT® robot (Titan Medical, Inc.; Toronto, ON, Canada). All of these new robotic systems are single-port systems with smaller robotic arms to provide improved access to difficult to reach locations (65). The value of these newer robots can be seen in the recent trial (33) of the da Vinci Single Port robot, a flexible single-arm device. That trial confirmed the capacity of this robot to safely provide access to the nasopharynx, oropharynx, larynx, and hypopharyngeal regions.

Other advances currently being studied include augmented reality (AR), which provides real-time image-guidance with navigational cues and representations of key anatomical structures overlaid on the operative field. In addition, cone beam computed tomographic (CBCT) angiography has been proposed to provide image-guidance for the dissection of critical vascular landmarks and resection of base-of-tongue neoplasms with adequate margins (66). One of the main issues in some tumor locations, particularly those in close proximity to critical blood vessels and key anatomical structures, is the lack of haptic sensation (67). This is why image guidance is so important, as it can provide visual data of the operative field which can be combined with AR to permit surgery even in areas previously thought impossible.

## Conclusions

In the span of a very short time—less than a decade—robotic head and neck surgery has transformed the management of the head and neck cancer, and it seems clear that the future of treatment for these cancers lies in a multimodal approach in which TORS is likely to play an important role. Nevertheless, it is important to keep in mind that the current indications for TORS are limited and long-term data on the safety and oncological outcomes are needed to better understand the true role of TORS in treatment of head and neck cancer. Nonetheless, the emergence of ever more advanced robotic instruments is expected to further expand the indications for and use of TORS to optimize oncological outcomes while preserving organ function and patient quality of life.

## Author Contributions

The author confirms being the sole contributor of this work and has approved it for publication.

### Conflict of Interest Statement

The author declares that the research was conducted in the absence of any commercial or financial relationships that could be construed as a potential conflict of interest.
